# Investigation of the Chromosome Regions with Significant Affinity for the Nuclear Envelope in Fruit Fly – A Model Based Approach

**DOI:** 10.1371/journal.pone.0091943

**Published:** 2014-03-20

**Authors:** Nicholas Allen Kinney, Igor V. Sharakhov, Alexey V. Onufriev

**Affiliations:** 1 Genomics Bioinformatics and Computational Biology, Virginia Tech, Blacksburg, Virginia, United States of America; 2 Department of Entomology, Virginia Tech, Blacksburg, Virginia, United States of America; 3 Department of Physics, Virginia Tech, Blacksburg, Virginia, United States of America; 4 Department of Computer Science, Virginia Tech, Blacksburg, Virginia, United States of America; Wake Forest University, United States of America

## Abstract

Three dimensional nuclear architecture is important for genome function, but is still poorly understood. In particular, little is known about the role of the “boundary conditions” – points of attachment between chromosomes and the nuclear envelope. We describe a method for modeling the 3D organization of the interphase nucleus, and its application to analysis of chromosome-nuclear envelope (Chr-NE) attachments of polytene (giant) chromosomes in *Drosophila melanogaster* salivary glands. The model represents chromosomes as self-avoiding polymer chains confined within the nucleus; parameters of the model are taken directly from experiment, no fitting parameters are introduced. Methods are developed to objectively quantify chromosome territories and intertwining, which are discussed in the context of corresponding experimental observations. In particular, a mathematically rigorous definition of a territory based on convex hull is proposed. The self-avoiding polymer model is used to re-analyze previous experimental data; the analysis suggests 33 additional Chr-NE attachments in addition to the 15 already explored Chr-NE attachments. Most of these new Chr-NE attachments correspond to intercalary heterochromatin – gene poor, dark staining, late replicating regions of the genome; however, three correspond to euchromatin – gene rich, light staining, early replicating regions of the genome. The analysis also suggests 5 regions of anti-contact, characterized by aversion for the NE, only two of these correspond to euchromatin. This composition of chromatin suggests that heterochromatin may not be necessary or sufficient for the formation of a Chr-NE attachment. To the extent that the proposed model represents reality, the confinement of the polytene chromosomes in a spherical nucleus alone does not favor the positioning of specific chromosome regions at the NE as seen in experiment; consequently, the 15 experimentally known Chr-NE attachment positions do not appear to arise due to non-specific (entropic) forces. Robustness of the key conclusions to model assumptions is thoroughly checked.

## Introduction

Unlike enzyme proteins, which usually adopt the same unique three-dimensional (3D) shapes in all cells, the conformational states of chromatin fibers are not nearly as compact and ordered; the basic principles governing these conformational states are only beginning to emerge through computation and experiment [1]–[10]. Just like in the case of many polymers, the states of folded chromatin in the cell nucleus are expected to depend on the “boundary conditions”, in this case the location and properties of the nuclear envelope (NE). For example, if an “unrestricted” random coil were the same length as the human genome, it would occupy a 3D space many times greater than the volume of a typical cell nucleus, implying that in reality boundary conditions restrict the polymer to a much smaller actual volume matter. General polymer physics arguments suggest that the conformational state of chromatin across cell types depends strongly on the chromosome to nucleus volume ratio, and thus, there may be different folding principles in different lineages e.g. human and yeast cells [3]–[6], [11], [12]. Indeed, recent computational studies have demonstrated that chromosome organization in the nucleus may strongly depend on the degree of (spherical) confinement [13]: increasing the degree of confinement mimicked the effect of increasing chromosome looping probability, reinforcing the idea that the boundary conditions of the nucleus matter. These [13], and the results of other studies [3], [14]–[20], have raised the possibility that the boundary conditions of the nucleus, chromosome topology, and non-specific (entropic) forces may be sufficient to account for the organization of chromosomes in the nucleus of some Metazoans. Furthermore, chromosome looping, potentially brought about by the degree of confinement [13], has been linked to gene expression levels; specifically, higher chromosome looping probability was associated with higher local chromosome density and lower transcriptional activity in a recent study [21].

In addition to including the NE in computational chromosome models; many studies now take into consideration the specific interactions of the chromosomes with the [5], [6]. For example, a recent model of the yeast nucleus recapitulated key features of 3D chromosome organization and incorporated both centromere and telomere attachment to the [5], [6]. However, the nature of these interactions with the NE remains unclear; other studies have suggested that non-specific and specific forces acting together position chromosomes in the nucleus [16], and a recent study demonstrated that non-specific forces alone may be sufficient to localize chromocenter and heterochromatin to the in *Arabidopsis* [17]. Regardless of mechanism, identifying regions of chromosome-nuclear envelope (Chr-NE) contacts and “anti-contacts” (regions which statistically avoid the NE) is important for their inclusion in future modeling studies and for determining the types of chromatin typically found at or away from the nuclear periphery, which is in turn important for better understanding of 3D-chromosome organization. Stated simply, the main goal of our study is to objectify the finding of Chr-NE attachments and characterization of their composition.

Earlier experiments [9] discovered 15 Chr-NE attachments, identified by their high probability of contact with the NE exceeding 66% in an ensemble of 24 nuclei. These 15 known Chr-NE attachments coincide almost exclusively with regions of intercalary heterochromatin – gene poor, dark staining, late replicating regions of the genome [22]. The seminal study has clarified the character of the most frequent NE attachments, but left several important questions unanswered. Does the 66% ad-hoc threshold used previously for discovering Chr-NE attachments reveal all of the Chr-NE attachments in *Drosophila* polytene chromosomes, too many, or too few? Using a more objective threshold here is important because the composition of chromatin inferred from the analysis of the Chr-NE attachments may change if too many or too few Chr-NE attachments are identified. The use of a more objective threshold may help reveal previously uncharacterized NE attachments in the old experimental data; if those attachments are indeed found, then what is their heterochromatic character? Finally, could pure geometric effects, such as confinement in a spherical nucleus, specific chromocenter arrangement, and the excluded volume of the chromosomes and nucleolus favor the placement of specific chromosome positions at the NE, and could these non-specific (entropic) forces alone position the 15 most significant Chr-NE attachments?

Our study is designed to address these and several other questions, while delivering to the community a computational model that can be used to complement experiments that study the 3D architecture of chromosomes. Here we use polytene chromosome from salivary gland nuclei of *D. melanogaster*, which is a well-established model for studying organization and function of the eukaryotic genome [23]–[26]. Each of the polytene chromosomes contains approximately 1024 chromosome replicas bundled together in parallel; thus the entire genome organization in a single nucleus becomes visible under a light microscope. This is a critical advantage over “regular” interphase chromosomes because it becomes possible to obtain full spatial information about the position of each individual polytene chromosome – its complete trace in 3D space. The study of polytene chromosomes has significant potential for general understanding of 3D genome organization because recent experiments revealed identical structural and functional organization of non-polytene and polytene chromosomes in fruit fly [27]–[30]. Moreover, the polytene chromosomes are estimated to occupy about a third of the nuclear volume [31]; this chromosome to nuclear volume ratio, which critically affects the over all 3D nuclear architecture [2], is the same in regular non-polytene nuclei [32], and is likely similar to the values characterizing human nuclei [3].

Experimental studies have identified several plausible biological roles and effects of Chr-NE contacts, such as maintenance of nuclear architecture and separation of the chromosome territories [33]–[37]. Despite their importance, experimental validation and analysis of Chr-NE contacts in most non-polytene interphase nuclei is difficult since regular interphase chromosomes and their NE contact sites cannot be visualized directly by standard techniques of light microscopy. Instead, Chr-NE contacts in non-polytene interphase nuclei are often identified by indirect methods with fluorescence *in situ* hybridization [38] or inferred using a DamID approach – a method based on detecting DNA methylation by a chimeric protein consisting of a chromatin protein fused with methyltransferase [39]–[41]. The drawback of fluorescence *in situ* hybridization is that only a small number of chromosome positions can be labeled; consequently, determining the complete folding pattern of the chromosomes in a single nucleus is nearly impossible. The drawback of using a DamID [41] approach is that methylation via methyltransferase can only be detected using an entire ensemble of cells; consequently, the stochasticity and cell-to-cell variability of the Chr-NE contacts is lost. In polytene chromosomes, 3D tracing experiments have been used [9], [31] to directly visualize chromatin folding and to identify Chr-NE attachments, but these studies typically involve small numbers of nuclei, which makes establishing statistical significance difficult. The model described in this study is used to improve the criteria for identifying statistically significant Chr-NE attachments, and consequently improve our knowledge regarding the type of chromatin found at or away from the NE.

The polytene chromosomes from *D. melanogaster* salivary glands have been extensively characterized in previous experiments [9], [42], [43]. We model each of the five largest chromosome arms of *D. melanogaster* as a random self-avoiding walk (SAW) under confinement; parameters of the model come from available experimental data. We validate our method of model building by quantifying the experimentally observed presence of chromosome territories and the absence of chromosome intertwining. The model answers three questions: Are there additional statistically significant Chr-NE attachment regions? If there are additional Chr-NE attachment regions, do they also correspond to heterochromatin? Does confinement of the polytene chromosomes in a spherical nucleus alone favor the positioning of specific chromosome regions at the as seen in experiment? Our model demonstrates that the geometric effects of chromosome confinement inside a spherical nucleus alone do not bring about specific Chr-NE attachments. We use our model to improve criteria for locating Chr-NE attachments. By applying our criteria to the data available from previous tracing experiments [9], [42], [43] we identify 33 new, previously unreported, but statistically significant Chr-NE contacts and 5 regions of anti-contact. The composition of these new Chr-NE attachments is discussed.

## Materials and Methods

### Model Building

#### Motivation

Our model incorporates *all* experimentally known parameters *D. melanogaster* polytene chromosomes *with the exception* of introducing specific Chr-NE attachments; in other words, the model is a Null model with respect to Chr-**NE attachment**. Essentially, the deviations between our Null model and experiment then reveal the positions of Chr-NE attachment from experimental data (this is the focus of the paper and is discussed extensively in what follows). We construct the Null model using an equilibrium based self-avoiding walk approach and introduce several modifications in order to recapitulate experiment. Some of these modifications likely introduce non-equilibrium features into our model; however, we stress that the fully modified model contains all the known features of the polytene nucleus from experiment except for specific Chr-NE attachments. For any other model the deviations from experiment would arise from multiple factors, not just the Chr-NE attachments. Regardless, we check that all model conclusions are robust to the non-equilibrium features that our model contains (discussed below).

#### Approach

**Figure 1:**
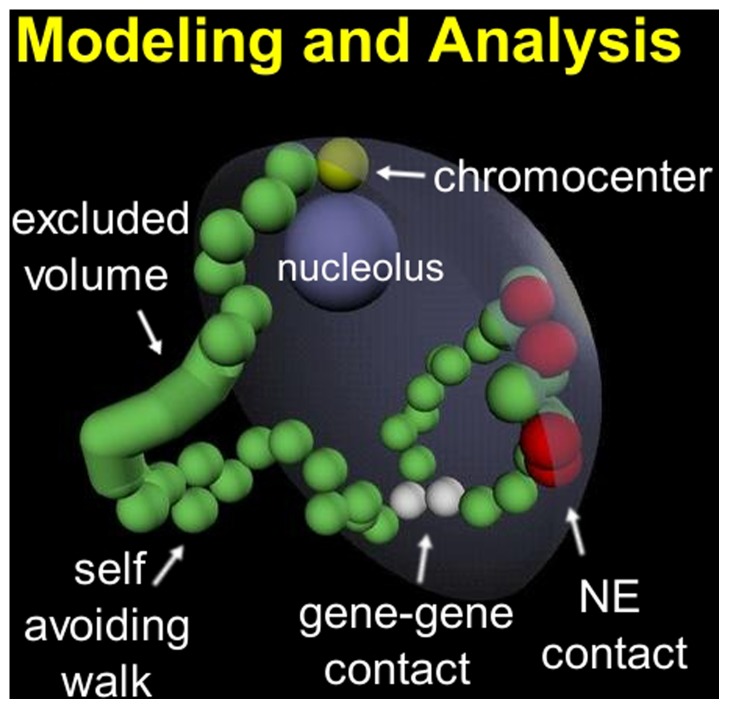
Computational model of equilibrium states of a *Drosophila* polytene nucleus. The five chromosome arms are represented by five random SAW chains under confinement (one SAW chain is shown). The chains are built simultaneously starting from the chromocenter (yellow). Contacts are beads within one micron of the NE (red); locus-locus contacts are beads within two microns of each other (white). Full excluded volume includes the cylinder connecting two adjacent beads.

The five largest chromosome arms of *D. melanogaster* salivary glands are modeled as beads-on-string [2], [4], [16], [44]–[46] and are represented as five random self-avoiding walks (SAWs) [47]–[50] (Figure 1). This approach is common in theoretical studies of 3D chromosome architecture, and has already been shown to recapitulate some properties of experimental ensembles of polytene chromosomes [51]. The sixth arm, chromosome 4, is not considered due to its negligible length. Experimental data for the chromosomes and the nucleus become realistic model parameters and constraints imposed during the construction of SAWs (see Text S1 for a complete derivation of all model parameters and constraints).

#### Modeling procedure

**Figure 2:**
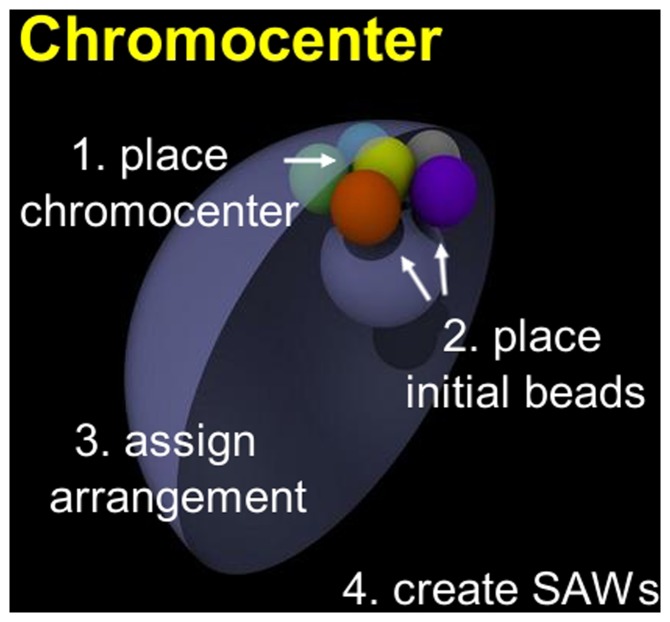
The first four steps of constructing the model nuclei. SAW = self-avoiding random walk.

**Figure 3:**
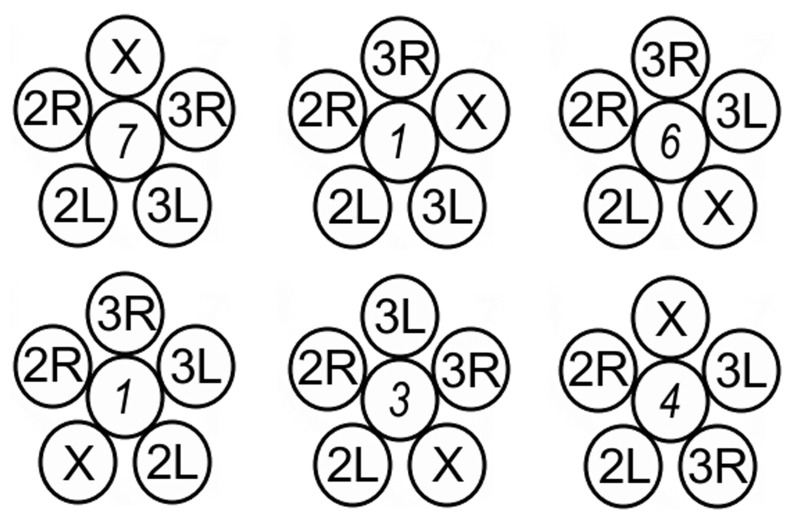
Relative number of chromocenter arrangements in 22 experimental nuclei [9]. Numbers in italic represent the total nuclei found with the corresponding arrangement of the chromosome arms.

One bead representing the chromocenter is placed adjacent to the NE (yellow bead in Figure 2) at the “north pole” of the nucleus. Then, five initial beads are placed, without overlapping, at random angular positions around the chromocenter (Figure 2); these five beads touch the chromocenter and NE, mimicking the experimental configuration of *D. melanogaster* chromocenter with the five chromosome arms extending outward. The arrangement of the five initial beads around the chromocenter bead is designed to match the relative proportion of chromocenter spatial arrangements seen in experiment [9] (Figure 3, details in Text S1). After assigning the chromocenter arrangement, SAWs are constructed using Rosenbluth algorithm [52] (i.e. SAW chains grow by addition of monomers in a “true” SAW fashion). We use a Rosenbluth algorithm for computational efficiency; for short chains this approach is a good approximation of self-repelling chains which are true equilibrium states of polymers [53]. This approach was recently used to generate densely packed SAWs in a study of protein folding [54]. Although our model is based on a SAW model, which is equilibrium by construction (to the extent that it approximates self-repelling chains), two non-equilibrium features are introduced to better represent experiment; these include the Rabl configuration of chromosomes and right-handed chromosome chirality.

It is known that most *D. melanogaster* polytene chromosomes conform to the Rabl type configuration [9]. This configuration is characterized by the predominant (80%) presence of the chromosome telomeres in the nuclear hemisphere opposite the chromocenter. It has been speculated that the Rabl configuration of chromosomes is a remnant of anaphase, which upon formation of Chr-NE attachments, may trap chromosomes in non-equilibrium configurations within the nucleus. However, the nature of Rabl configuration is not completely clear; an alternative possibility is that formation of Chr-NE envelope attachments trap chromosomes in a polarized configurations within the nucleus which remain polarized after reaching equilibrium. Rabl configuration was enforced in our models by *a posteriori* filtering of the generated ensembles of nuclei to achieve Rabl configurations in the final ensemble (details in Text S1). This *a posteriori* filtering introduces a non-equilibrium modification of the SAW’s forming the basis of our model and is intended to reproduce the Rabl configuration seen in experiment (see Figure S1); but, this does not necessarily imply that the experimental polytene chromosomes in the nucleus are non-equilibrium for the reasons stated above.

Studies that trace the path of each chromosome arm in *D. melanogaster* salivary gland nuclei have observed a disproportionate amount (2∶1) of right handed twist compared to left handed twist [9]. We enforce right handedness in our simulated chromosomes during construction of the SAWs: it is twice as likely for a new bead to be accepted if it forms right handed chirality rather than left handed chirality (details in Text S1). This introduces a second non-equilibrium modification of the SAWs that form the basis of our model; the modification is intended to reproduce the chirality seen in experiment. This modification also does not imply that the experimental polytene chromosomes in the nucleus are non-equilibrium; the right-handed chirality seen in experiment may be equilibrium with dihedral potentials that are currently unknown. To address the question, one has to go beyond the current model.

**Figure 4:**
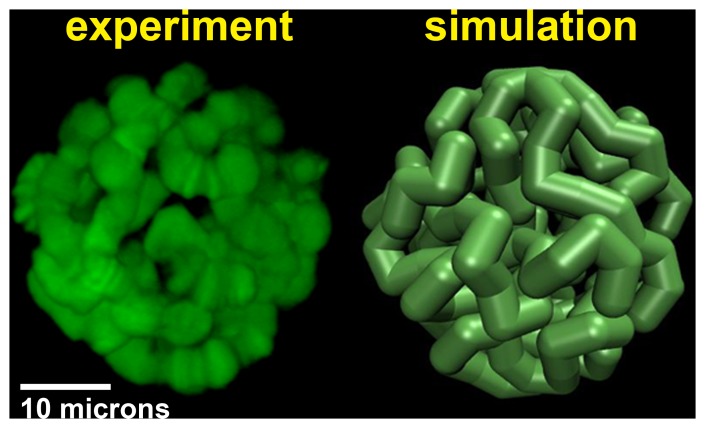
Representation of a simulated model nucleus (right) compared to experiment (left).

A single step in growing the SAWs consists of simultaneously picking a random direction in 3D space to extend each model chromosome arm, adding the five new beads, and checking for violation of model constraints such as excluded volume (no bead overlap) and right-handed chromosome chirality. If no model constraints (see below) are violated, then the new beads are accepted and the model chromosome arms continue growing. In the case of rejecting the new beads, the step is repeated with new random directions in 3D space. The avoidance of perpetual SAW rejections is accomplished with two backtracking parameters, BT_1_ and BT_2_, that tally the number of SAW rejections. A single bead backtrack is made after BT_1_ = 2000 failed SAW additions, followed by its resetting; a 5 bead backtrack is made after BT_2_ = 6000 failed SAW additions, followed by its resetting. The above process is repeated until model completion (see Figure 4). This simple technique allows us to easily impose preferred right handed twist, chromocenter arrangement, and chromosome confinement which would be more difficult to enforce using alternative model building techniques [2], [3], [55]. The main conclusions of this work are insensitive to the choice of BT_1_ and BT_2_, see below. We chose the manifestly symmetric SAW construction procedure (at each step the beads for all the chromosomes are added simultaneously) because there is no biological evidence that suggests a spatial symmetry breaking between the chromosomes. That is a conceivable alternative procedure in which a certain chromosome is fully built first, followed by other(s) would be less justified biologically.

#### Robustness of major conclusions to model details

The general SAW approach introduced here to model polytene chromsomes was validated in several previous studies in similar contexts [2], [3], [5], [6], [51]. To improve biological realism of our model, we have introduced several additional features to the basic procedure. For simplicity we construct our SAW’s using an unweighted Rosenbluth algorithm [52]. It is reiterated that our final ensemble reproduces all known features of experimental polytene chromsomes in the nucleus without enforcing specific Chr-NE attachments; consequently, the deviations between our model and experiment must stem from Chr-NE attachments alone. Regardless, we have checked explicitly that the key model conclusions are robust to all non-equilibrium SAW modifications introduced in our model. To this end, two variations of our SAW were considered: (1) *fully modified SAW* – with Rabl configuration, right-handed chirality, and chromocenter arrangement designed to recapitulate all features of experimental nuclei with the exception of Chr-NE attachments. This is the main model used in this work. In addition, we have considered: (2) *unmodified SAW* – which does *not* introduce Rabl configuration, right handed chirality, or chromocenter arrangement, and so is equilibrium to the extent that our chain growing algorithm approximates self-repelling chains. Both variations of our SAW model lead to the same main conclusions (see Table S1 and Table S2). To enforce constraints in our models (spherical boundary and excluded volume) we use a simple backtracking procedure controlled by two parameters, *BT*_1_ and *BT*_2_ (see methods) that determine when a single bead and 5 bead backtrack is made respectively during the construction of the SAW. Unlike all other parameters of the model, the values of these two parameters do not come from experiment. To verify robustness of the key conclusions to the specific choice of BT_1_ ≫ 1 and BT_2_ ≫ 1, we used a third variation of our SAW approach: *fully modified SAW with BT_1_ = 1000 and BT_2_ = 3000* – with backtracking parameters reduced by a factor of 2. This variation of our model also yielded the same main conclusions (see Table S1); computational complexity prohibited testing model robustness in the entire BT_1_–BT_2_ parameter space.

#### Derivation of model parameters and constraints from biological data

See Text S1.

### Simulations

**Figure 5:**
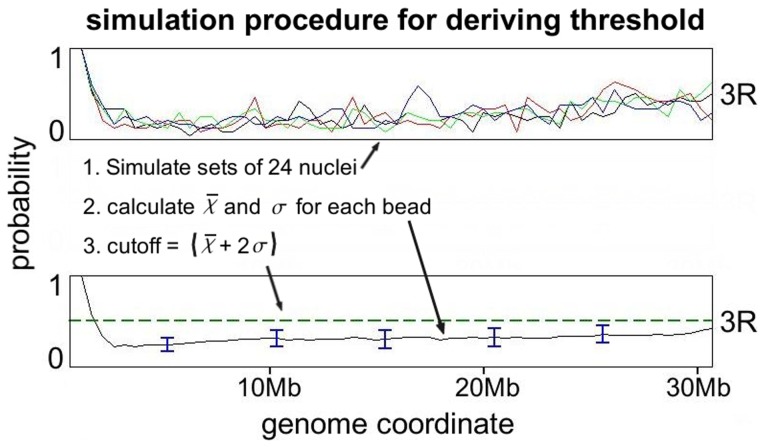
Procedure for deriving statistically significant threshold for identifying Chr – NE contacts in chromosomes tracing experiments (only chromosome 3R is shown for clarity). 96 sets of 24 nuclei were simulated (without enforcement of Chr-NE contacts). NE contact frequency for each chromosome position is plotted as a “contact frequency profile”; profiles from 4 independent simulations are exemplified in the top panel. The mean (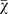) contact frequency and the standard deviation obtained from these simulated tracing experiments are used to set a threshold for identifying statistically significant Chr-NE contacts (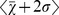) and anti-contacts (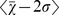).

**Figure 6:**
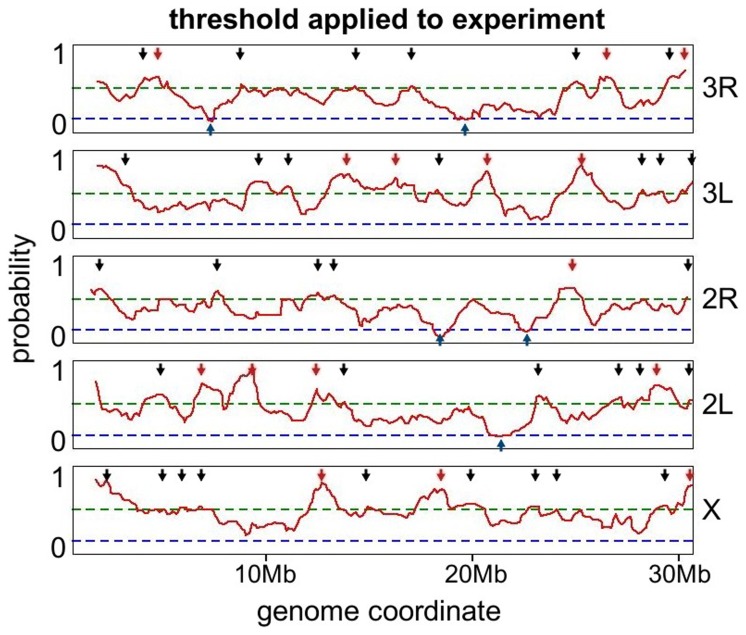
High frequency and sub-high-frequency NE-contacts at a new threshold (green dashed line) of 50.5% (2σ) significance. Red line – experiment [9]. Red arrows are the original 15 contacts identified in [9]. Black arrows are the additional contacts which are statistically significant according to our simulations. Blue arrows are significant regions of anti-contact – contacts that occur below the threshold (blue dashed line) of 14.3% (2σ) significance.

A previous experiment [9] estimated Chr-NE attachment probability for each chromosome position in 24 nuclei; each nucleus represented a single snapshot of the true state of the chromatin – a conformational ensemble of the five chromosomes. In this previous experiment (1) 15 Chr-NE attachments were defined by setting an ad hoc threshold of >66% probability of observed contact with the NE. We use our model to essentially simulate a large, statistically significant number of these same tracing experiments also with 24 nuclei, but without specific Chr-NE attachments. Upon simulating 96 repeated tracings of 24 experimental nuclei (four shown in Figure 5), we calculate the mean, 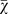, and the standard deviation, 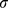, in contact frequency for each bead. It is unlikely to observe beads in our simulations (24 nuclei) with frequency of NE contact greater than 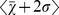. We identify 48 Chr-NE contact frequencies above this threshold (green line Figure 5 and 6) in previous experimental data [9]. The only difference between our model and experiment is the presence of specific Chr-NE attachments in the latter, and so it is statistically highly unlikely that the 48 experimentally determined Chr-NE contact frequencies are above the 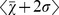 threshold due to pure chance (black and red arrows in Figure 6). By definition, approximately 2.5% of Chr-NE contact frequencies were above this threshold in our Null model that contains no specific Chr-NE attachments; thus, a lower statistical threshold would run the risk of identifying more “false positives” in experimental data while higher levels of significance would overlook the true Chr-NE attachments in experiment. We checked that 96 repeated simulations are enough to yield a reproducible 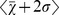 threshold, see Table S1.

Using this same analysis, a threshold set at 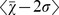 was used to establish statistical significance for regions of *anti-contact* - regions which statistically avoid the NE. With this definition, we identified statistically significant Chr-NE anti-contacts in previously published experimental data (blue line in Figure 6) [9]. A large number of model nuclei (96×24 = 2304 model nuclei) were needed to simulate these repeated tracing experiments because a new set of 24 nuclei was used for each simulated experiment.

### Analysis of the Simulations

#### Simulated tracing experiments

For a single chromosome arm in a model nucleus, an array is formed with entries for each bead in the chromosome arm. For every bead an entry of 1 is recorded in the array if contact occurs with the NE. A frequency profile (Figure 5) is formed by averaging corresponding entries in 24 arrays, this being the same number of nuclei that was used in a previous experiment [9]. In our study this procedure is repeated 96 times, simulating the outcome of 96 chromosome tracing experiments involving 24 nuclei each; the average contact frequency, 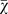, and standard deviation, 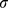, for each bead in these 96 simulated tracing experiments is calculated. The standard deviation of these simulated chromosome tracing experiments provides a measure of how contact frequencies for a single set of 24 nuclei *may* change for repeated experiments.

#### Chromosome territory index

**Figure 7:**
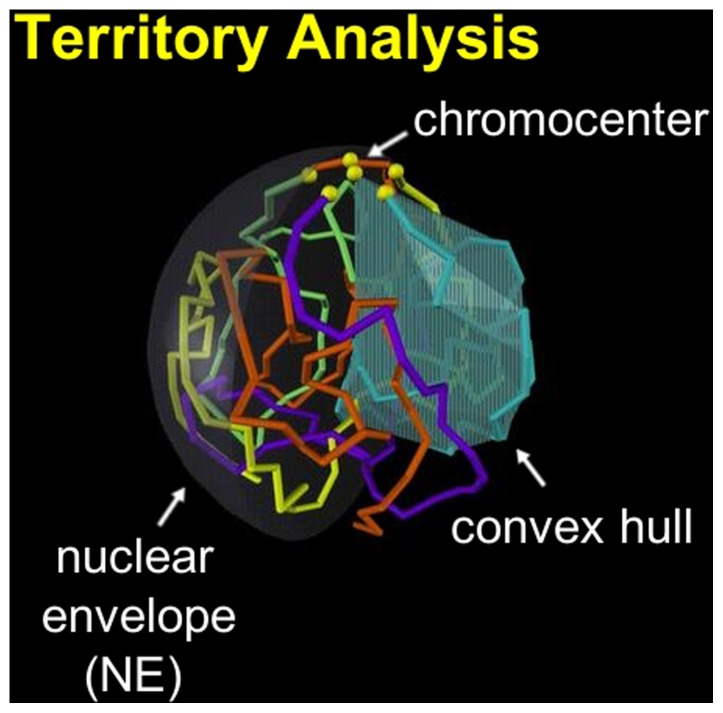
The territory index of a chromosome is defined as the percent of its beads found inside the chromosome’s own convex hull. Example: light blue chromosome.

Chromosome territories [1], [2], [56] are assessed by quantifying how effectively one chromosome excludes other chromosomes from the volume it occupies in the 3D space. There is no universally accepted definition of chromosome territory, and, to the best of our knowledge, there is no mathematically rigorous one either. Our definition of the territory is similar in spirit to the construct used to define Kolmogorov-Sinai entropy. We begin by calculating the convex hull for a single chromosome, Figure 7 (we use MATLAB [57]), this is the minimum volume that includes all the chromosome’s points (bead centers) inside a convex polyhedron. In general, each convex hull contains its own chromosome, and may also encompass some points belonging to other chromosomes. A fully “territorial” chromosome is one whose convex hull does not contain points from any other chromosomes while a less “territorial” chromosome is one whose convex hull contains some points from other chromosome. We define the chromosome territory index as the fraction of points inside a convex hull that belong to the chromosome used for its construction; for example, the fraction of light blue chromosome points inside the light blue convex hull shown in Figure 7. Under this definition the maximum territory index is 1. The minimum territory index for a chromosome depends on how many beads the chromosome’s convex hull can possibly accommodate; different chromosomes have a different minimum territory index. To establish this minimum territory index for a chromosome arm having 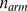 beads, the 3D chromosome configuration having a global maximum convex hull volume under spherical confinement, 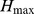, is found (Figure S2). The minimum territory index for the chromosome is then given by 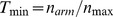, where 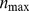 is the maximum number of beads that 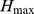 can accommodate.

We approximate 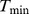 for each chromosome of fruit fly by finding the chromosome configuration that corresponds to 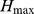 (see Figure S2 for details). For each chromosome, the volume of this 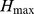 (Figure S2 and Figure S3) was found to exceed the total volume of all 248 beads in our model nucleus (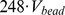) implying that a fully “anti-territorial” chromosome is one whose convex hull contains all 248 beads from itself and all other chromosomes in our model; thus, for our model 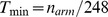. Using these definitions, the lowest territory index ranges from.18 to.24 depending on the chromosome.

#### Test for chromosome intertwining

Chromosome intertwining is an intuitive concept that can be rigorously assessed by attempting to separate model chromosomes by a translation in 3D space: if the two chromosomes can be separated in this manner, we call them non-intertwining. We begin by selecting the backbone of two chromosomes (including centromere) from a model nucleus; the backbone of a model chromosome consists of the line segments connecting the centers of each bead. A 35 micron long direction vector is then chosen to translate the backbone of one model chromosome; (the translation vector is longer than the diameter of the nucleus). If the two backbones cross during this translation then a new direction vector is picked. A total of 162 different direction vectors are tested in this manner, with unit vectors that uniformly cover the 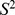 space (spherical surface). If it is possible by one of these translations to separate the chromosome backbones, then the two chromosomes do not intertwine. The amount of intertwining in an ensemble is quantified by calculating the percent of all chromosome pairs in the ensemble that intertwine.

## Results

### Validation of the Model

#### Chromosome to nucleus volume ratio

The calculated chromosome to nuclear volume ratio is .30 in our model, close to the experimentally measured ratio of .34 in *D. melanogaster* [31]. The difference which arises from coarse graining of the chromosomes is likely to be within the margin of error of the experiment.

#### Chromosome territories

Experimental, qualitative descriptions of *D. melanogaster* polytene nuclei have established that chromosomes form territories, “analogous to the sections of a grapefruit” [9], [58] (Figure 7). The average territory index per chromosome in our model is .650 out of the highest possible value of 1.0 (see the precise definitions in “Methods”); the computed territory indexes are significantly higher than the smallest possible territory index in fruit fly, which ranges from.18 to.24, depending on the chromosome. The comparison confirms that our model chromosomes are indeed “territorial”. Thus, no additional, territory-specific *a posteriori* filtering was needed within our model to recapitulate this critical feature of chromosomes seen in experiment. We interpret this as validation of our modeling approach; we further checked the robustness of our modeled chromosome territories to the non-equilibrium modifications of our SAWs. Specifically, an unmodified SAW model (also described in robustness section) without right-handedness, Rabl orientation, or preferred chromocenter arrangement had an average territory index per chromosome of .651. Thus, non-equilibrium considerations may not be needed to account for the territorial property of polytene chromosomes in *D. melanogaster* salivary gland nuclei. Incidentally, we noted that a subjectively (visually) “territorial” model chromosome does not imply a chromosome territory index of 1; for example, a territory index of .650 has a qualitative description similar to the qualitative descriptions of previous experiments [9], (see Figure S4). The degree of objectivity and rigor that we have introduced by our definition of chromosome territory may therefore be useful in analysis of both experimental and modeled chromosomes.

#### Intertwining

Experimental, qualitative descriptions of *D. melanogaster* polytene nuclei have established that salivary gland chromosome arms do not intertwine [9], [31], [58]. We calculated the percent of non-intertwining chromosome arms (see methods) in our models. This analysis suggests that the percent of non-intertwining chromosome arms approaches 95% using our modeling method, (see details in Figure S5). We interpret this agreement with experiment as an indication of the strength of our model. The virtual absence of chromosome intertwining within our model was also robust to the non-equilibrium modifications of the model; approximately 95% of chromosomes in an unmodified model (described in robustness section) were also non-intertwining. In addition, we noted that our test for chromosome intertwining is highly sensitive; some chromosomes which may subjectively (visually) be identified as “non-intertwining” still narrowly failed the rigorous test (see Figure S6).

#### Scaling properties of the generated SAWs

The end-to-end length of our simulated SAWs in free space is described by 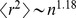, where *r* is chain end-end length and *n* is number of beads; this is in good quantitative agreement with theoretical results that give a range of 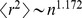 to 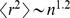 [59]–[63]. When we exclude the volume of the bond between nearest neighbor beads (Figure 1), the end-end length of our SAW’s in free space is described by 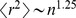 (Figure S7).

### Additional High Frequency Contact Positions are Suggested by Simulation

#### Improved criterion for identifying chromosome – NE contacts

A contact frequency of .505 was on average (not including the centromere) two standard deviations above the mean for beads in our model (Figure 5 bottom panel), this value defined an objective threshold used to identify additional Chr-NE contact positions in the experimental data for a single set of 24 nuclei. Although this amounts to a lowering of the.66 frequency threshold originally use to identify the 15 Chr-NE attachments in (1), we stress that our model is intended to *improve* the threshold used to identify Chr-NE attachments not to simply lower it; an *ad hoc* threshold that is too high or too low could lead to an altered composition of chromatin at the NE and influence our understanding of Chr-NE attachment formation.

All peaks above our improved threshold were identified in the experimental data (Figure 6); nearby peaks also above the threshold were only considered if they were further away than the Kuhn length (3.1 microns) from neighboring peaks, this being the length over which there is no directional correlation in the chromosome fiber. We refer to the new Chr-NE contacts revealed as “sub-high frequency” to distinguish from the 15 previously reported “high frequency” Chr-NE contacts from [9].

#### Composition of newly identified chromosome–NE contact positions by chromatin type

**Figure 8:**
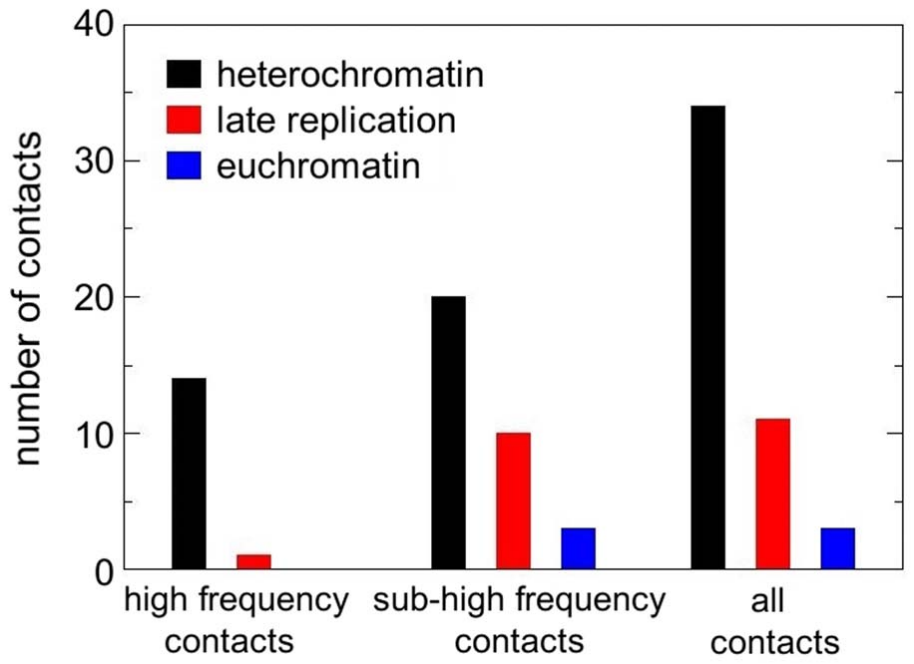
Composition of Chr-NE contacts in interphase polytene chromosomes of *D. melanogaster*. High frequency contacts were identified previously in experiment (left set of bars). New sub-high frequency contacts identified by our model (center). All contacts combined (right).

Most of the positions we identify are located in regions of the chromosome corresponding to intercalary heterochromatin–dark staining compact regions of the chromosome [64]. In total, we identify 33 additional Chr-NE contacts, 20 of which are intercalary heterochromatin and 3 euchromatin; the additional 10 Chr-NE contacts display some properties of heterochromatin by being late replicating regions [22] (Table S3). We classified a chromosome region as intercalary heterochromatin if it contained a site of late replication and localization of antibodies against SuUR (Suppressor of UnderReplication) protein in wild-type flies [22]. We classified a chromosome region as a region of late replication if it contained a site of late replication in wild-type flies or a site of localization of antibodies against SuUR protein in SuUR 4x flies, which have two additional *SuUR* ^+^ doses [22]. Of the 20 intercalary heterochromatin regions, 6 are regions of under-replication, which is typical to the large bands of intercalary heterochromatin [29]. Experiments have already demonstrated that the 15 most significant Chr-NE contacts in *D. melanogaster* are almost exclusively heterochromatic. Our results (Figure 8) suggest that affinity for the NE can change gradually, with the highest affinity for the NE almost *exclusively* possessed by intercalary heterochromatin, and the next highest affinity for the NE *mostly* a property of intercalary heterochromatin. The presence of 3 euchromatic regions in our set of 33 sub-high frequency contacts suggests that it is not necessary for a chromosome region to be heterochromatic in order to possess *some* affinity for the NE. We stress that this result is based on the known biological parameters of *D. melanogaster* polytene chromosomes.

#### Composition of contact positions with strong aversion for the NE

We applied the same analysis to identify positions of anti-contacts, which we define as chromosome regions that have significantly low probability to form a Chr-NE contact. A contact frequency of .143 was on average two standard deviations below the mean for any bead in our model; this defined a threshold used to identify anti-contacts. Five anti-contact regions were found below this threshold: 27B, 55E, 52F/53A, 85E, 93F (Figure 6). Three of these regions are euchromatic (55E, 52F, 85E), two are regions of late replication (27B, 93F), and one (52F/53A) is at the boundary between euchromatin and heterochromatin. The highly significant anti-contact at the border of regions 52F/53A may suggest that heterochromatin is not sufficient for formation of a Chr-NE contact. Thus at 1 Mb resolution, identifying a chromosome region as late replication, and possibly heterochromatin alone may not be completely sufficient to determine if a chromosome region will form a contact with the NE or if it will likely avoid it.

### Experimental Chromosome-nuclear Envelope Contact Positions Appear Non-random

A simple corollary follows from our computational modeling used primarily to reveal statistically significant Chr-NE attachments in experimental tracing data: because Chr-NE attachments are *not enforced* in our model nuclei (except for the chromocenter) these models should also reveal whether geometric effects alone (spherical confinement, presence of nucleolus, chromocenter arrangement, etc) predetermine which chromosome positions are in contact with the NE. When we average over 96 simulated chromosome tracing experiments, each bead in each chromosome in our model has approximately an equal chance to contact the NE (exemplified for 3R in Figure 5 bottom panel). This result shows that a bead’s purely geometric positioning in a model chromosome under spherical confinement has no effect on the affinity or aversion of that bead for the NE. Experiments [9] suggest, however, that 15 regions on the fruit fly chromosomes have a much higher affinity for the NE than the average. Statistically, it is virtually impossible for these 15 experimental Chr-NE contacts to arise in their corresponding beads due to pure chance in 24 model nuclei; thus, we conclude that, to the extent that our model represents reality, the 15 experimental Chr-NE contacts must have intrinsic affinity for the NE, unrelated to their pure geometric position along the chromosome.

## Discussion

### The Model

The random self-avoiding walk (SAW) is a classic, widely used approach to modeling polymers [4]–[6], [65]–[68]. Variations of the SAW were employed in studies that model 3D chromatin organization, and were shown to accurately capture average locus-to-locus distances [2], [4], [49], [51], [69]. Several models based on variations of a SAW have already been used to successfully explain the structural features of chromosome 2L in *D. melanogaster* polytene chromosomes [51]. These previous models used a variety of strategies to explain 3D chromosome structure: strategies which included incorporating the Rabl configuration of chromosomes and generating the SAWs under confinement [4], [51]. In this work we capitalize on the earlier successes of the SAW-based approaches to build a more realistic model of the 3D architecture of chromosomes in interphase nuclei - our model incorporates only the known parameters on *D. melanogaster* polytene nuclei, no fitting parameters are introduced. In addition, our model creates entire ensembles of nuclei which realistically describe the cell-to-cell variations in chromatin folding. The application of our model to the analysis of chromosome tracing experiments offers a new valuable tool. We have applied the model to a previous tracing experiment of 24 *D. melanogaster* salivary gland nuclei [9]; however, our model can easily be extended to the analysis of tracing experiments involving a different number of nuclei. In addition, our model can easily be applied to polytene chromosome of different cell types by reconfiguring the model with the corresponding parameters from experiment.

Chromosome territories, in which each chromosome occupies a distinct sub-volume of the cell nucleus, have been observed in many experiments including both polytene and non-polytene chromosomes [9], [31], [56], [70]–[74]. Recent simulations have demonstrated that non-specific entropic forces may play a significant role in establishing and maintaining chromosome territories [14], [19], [75], and it has been suggested [14] that this entropic effect stems from long flexible polymers having access to more chain configurations if they remain separate in distinct domains, rather than tangling together. This entropic effect has been shown to depend on the degree of confinement [76] and the presence of chromosome loops [14], which may also arise due to non-specific forces [14]. These arguments essentially assume that the chromatin reaches the state of thermodynamic equilibrium on the experimentally relevant time-scales. On the other hand, it has been argued that equilibrium configurations of human interphase chromosomes would not display territories and that territory formation is best explained by a non-equilibrium fractal globule [1], [2]. The prediction of chromosome territories in our model is robust to the non-equilibrium modifications we make to the underlying, essentially equilibrium, SAW model. This robustness suggests that non-equilibrium considerations may not be necessary to explain territories seen in polytene chromosome in fruit fly nuclei. Given that chromosome territories appear to be a generic feature of many genomes including human, our intuitive, yet mathematically rigorous and easily computable definition of the territory should be of interest as well.

### New Chr-NE Contact Positions

Previous experiments [9], [31] identified the 15 polytene chromosome positions with the highest probability to contact the NE; 14 of these corresponded to regions of intercalary heterochromatin. With the aid of our computational model we re-analyzed the experimental data and presented several important new results. First, our model provides a method to objectively define a Chr-NE contact or anti-contacts; these objective criteria are based only on robust statistical properties of polymer ensembles, the known parameters of *D. melanogaster* polytene chromosomes, and geometric dimensions of the *D. melanogaster* polytene nucleus. This analysis has led to identification of 33 new Chr-NE contacts, of which 20 are heterochromatic, 10 are late replicating, and 3 are euchromatic. This result suggests that affinity for the NE is not a discrete property; the most significant Chr-NE contacts may be *exclusively* heterochromatin [9], with less prominent contacts composed of *mostly* heterochromatin. We put forward a testable hypothesis that it is local density of heterochromatin that may determine the propensity to form Chr-NE contacts. Three of the Chr-NE contacts we identify are euchromatin suggesting that it may not be necessary for a chromosome region to be heterochromatic in order to have some affinity for the NE. We found 5 regions of anti-contact (avoiding NE): 2 euchromatic, 2 late replicating, and 1 at the boundary between a euchromatin and heterochromatin region. This shows that late replication and possibly heterochromatin may not be sufficient to place a chromosome region in contact with the NE.

In non-polytene interphase chromosomes, pericentric and intercalary heterochromatin has been shown experimentally to possess a mechanism of localization to the NE, specifically, by lamin [29], [39], [77]–[80]. A previous study of 24 polytene nuclei found that 14 NE contacts are composed of intercalary heterochromatin and one is a late replicating region [9]. A following study confirmed 12 contacts (at 9A, 12DEF, 22A, 33A, 35A, 36C, 57A, 64D, 67D, 83D–84A, 98C, 100AF) and identified four more NE-contacting sites at 97A, 19DE, 60EF, and 61AB [43]. Interestingly, these four contacts were also identified as sub-high frequency contacts in our study, and all four include regions of intercalary heterochromatin. Our study identified a total of 48 significant contact sites, 45 possessing properties of heterochromatin/late replication regions and 3 possessing properties of true euchromatin (Table S3). Thus, our results are consistent with previous experiments, but also suggest that intercalary heterochromatin (at 1 Mb resolution) is not completely necessary or sufficient for the formation of a Chr-NE contact; however, it may be necessary for formation of Chr-NE contacts at the highest level of significance [9].

A genome-wide study of DNA-lamin binding in embryonic cells using DamID has shown significant correspondence to polytene Chr-NE contacts in larvae [39]. This study has also indicated that lamin binding is linked to a combination of several features including late replication, large size of intergenic regions, low gene expression status, and the lack of active histone marks, suggesting that a combination of cell-type dependent and independent factors may influence NE association. Furthermore, this study [39] reported that when the c-terminal, nuclear membrane binding portion of lamin protein was deleted (referred to as Lam Δ^CaaX^) there was a negative correlation between Lam Δ^CaaX^ and polytene chromosome NE association; consequently, it was suggested that Lam Δ^CaaX^ may co-localize with genes that have an aversion for the NE. Both of these results are consistent with our findings: that late replication or heterochromatin alone may not be sufficient to bind a chromosome locus to the NE and that some regions of the chromosome can be preferentially located at the nuclear interior.

Another study [29] has compared localization of lamin-associated domains identified in DamID experiments [81] and 60 regions of intercalary heterochromatin. Interestingly, the overlap was far from complete: 6 regions of intercalary heterochromatin showed no overlap with any of the lamin-associated domains, and one region of intercalary heterochromatin encompassed five separate lamin-associated domains. Complete overlap was observed for 4 regions of intercalary heterochromatin [29] supporting our conclusion that intercalary heterochromatin is not completely necessary or sufficient for the formation of a Chr-NE contact.

### Overall Conclusions

The recently discovered correspondence between the organization of polytene and non-polytene chromosomes of *D. melanogaster* [28] has revived interest in using polytene chromosomes to study the 3D organization of the genome. Chromosome tracing experiments, as demonstrated in several classic studies [9], [31], [42], [82], can be used to reconstruct the 3D organization of polytene chromosomes; however, these types of experiments still remain bottlenecked by the labor required to trace even a small ensemble of nuclei. Our study shows that experimentally parameterized computational models can assist studies of experimentally reconstructed nuclei. Our computational models complement a previous experiment [9] by revealing 33 new Chr-NE contacts and 5 anti-contacts; most of the 33 new contacts have properties of heterochromatin. However, the intercalary heterochromatic regions in *D. melanogaster* number more than 100 [22] and the complete rules for chromosome positioning with respect to the NE remain undiscovered. Further experiments may reveal additional Chr-NE contacts corresponding to the remaining regions of heterochromatin, or perhaps, additional contacts composed of both heterochromatin and euchromatin. The composition of contacts and anti-contacts in this study suggest a conclusion similar to that in previous studies [39]: that the placement of chromosomes at or away from the NE does not depend exclusively on chromatin type and a more complicated set of rules governs the formation of Chr-NE contacts. Importantly, our computational modeling indicates that confinement of chromosomes in a spherical nucleus alone does not favor the positioning of specific chromosome regions at the NE as seen in experiment.

## Supporting Information

Figure S1 **A posteriori filtering to achieve in the final ensemble 80% of telomeres in the hemisphere opposite the chromocenter as seen in experiment.**
(TIF)

Figure S2 **The maximum volume of chromosome convex hull under confinement.** The convex hull volume of a chromosome is maximized using a pivot algorithm [2]. Random rotations of chromosome segments are preformed, rejecting those that do not increase the convex hull volume. Iterations are preformed until numerical convergence is achieved. A maximum convex hull for chromosome 3R under confinement (left) is shown next to a model nucleus (right).
(TIF)

Figure S3 **The maximum volume of chromosome convex hull in free space.** In free space the maximum convex hull is larger than the entire nucleus.
(TIF)

Figure S4 **Simulated nuclei with average territory index per chromosome .65.** The average territory index per chromosome over all simulated nuclei we generated was .65 (see methods), examples of single model nuclei with this territory index are shown above. The standard deviation of the territory index per chromosome was .04.
(TIF)

Figure S5 **Convergence of the non-intertwining frequency between pairs of chromosomes as the number of test directions for spatial separation is increased.** Shown is frequency of non-intertwining depending on the number of direction vectors tested (methods); this suggests that as the number of test directions increases the frequency on non-intertwining chromosomes in our models approaches 95%.
(TIF)

Figure S6 **Examples of model chromosomes that intertwine.**
(TIF)

Figure S7 **Scaling of self avoiding walks.** Each data point represents the square end-end length averaged over 1000 self avoiding walks. This averaging was repeated for self avoiding walks ranging from 50 monomers to 150 monomers. To capture the thickness of the chromosomes a cylinder of excluded volume was placed around the bond between nearest neighbor beads. Scaling with and without this extra excluded volume is shown above. Least square regression lines are shown for each set of points.
(TIF)

Table S1 **Robustness of thresholds to model details.**
(DOCX)

Table S2 **Robustness of territories and intertwining to model details.**
(DOCX)

Table S3 **Classification of chromosome-nuclear envelope contacts by chromatin type.**
(DOCX)

Text S1 **Derivation of model parameters and constraints from biological data.**
(DOC)

Text S2 **Robustness of threshold used to identify Chr-NE attachments.**
(DOC)

Text S3 **References.**
(DOC)
