# Supplementary figures and images for: Investigation of the Chromosome Regions with Significant Affinity for the Nuclear Envelope in Fruit Fly – A Model Based Approach
Source: PLoS One. 2014 Mar 20;9(3):e91943. doi: 10.1371/journal.pone.0091943 (PMC3961273; doi:10.1371/journal.pone.0091943)

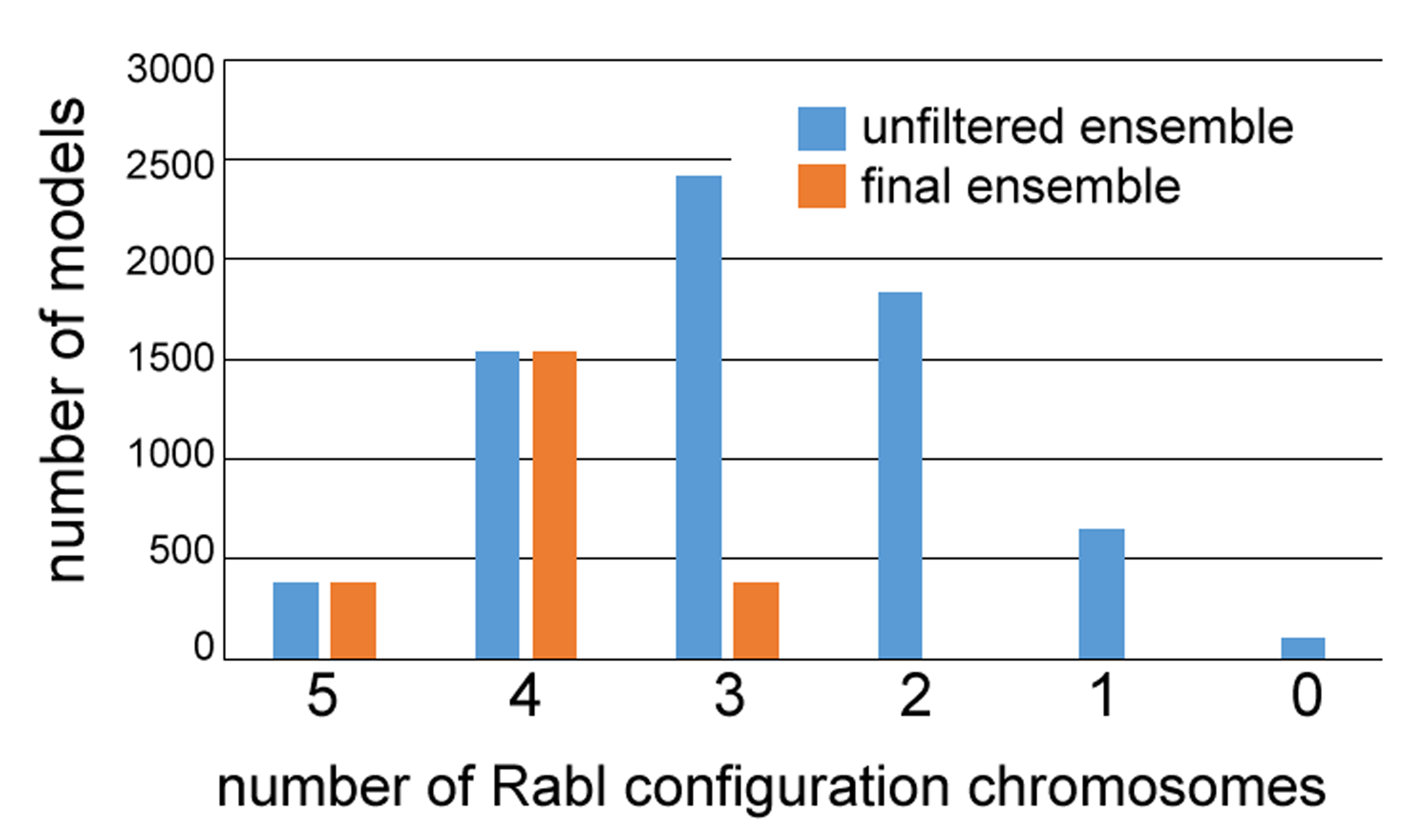

Supplement: Figure S1 — A posteriori filtering to achieve in the final ensemble 80% of telomeres in the hemisphere opposite the chromocenter as seen in experiment. (TIF) [file pone.0091943.s001.tif]

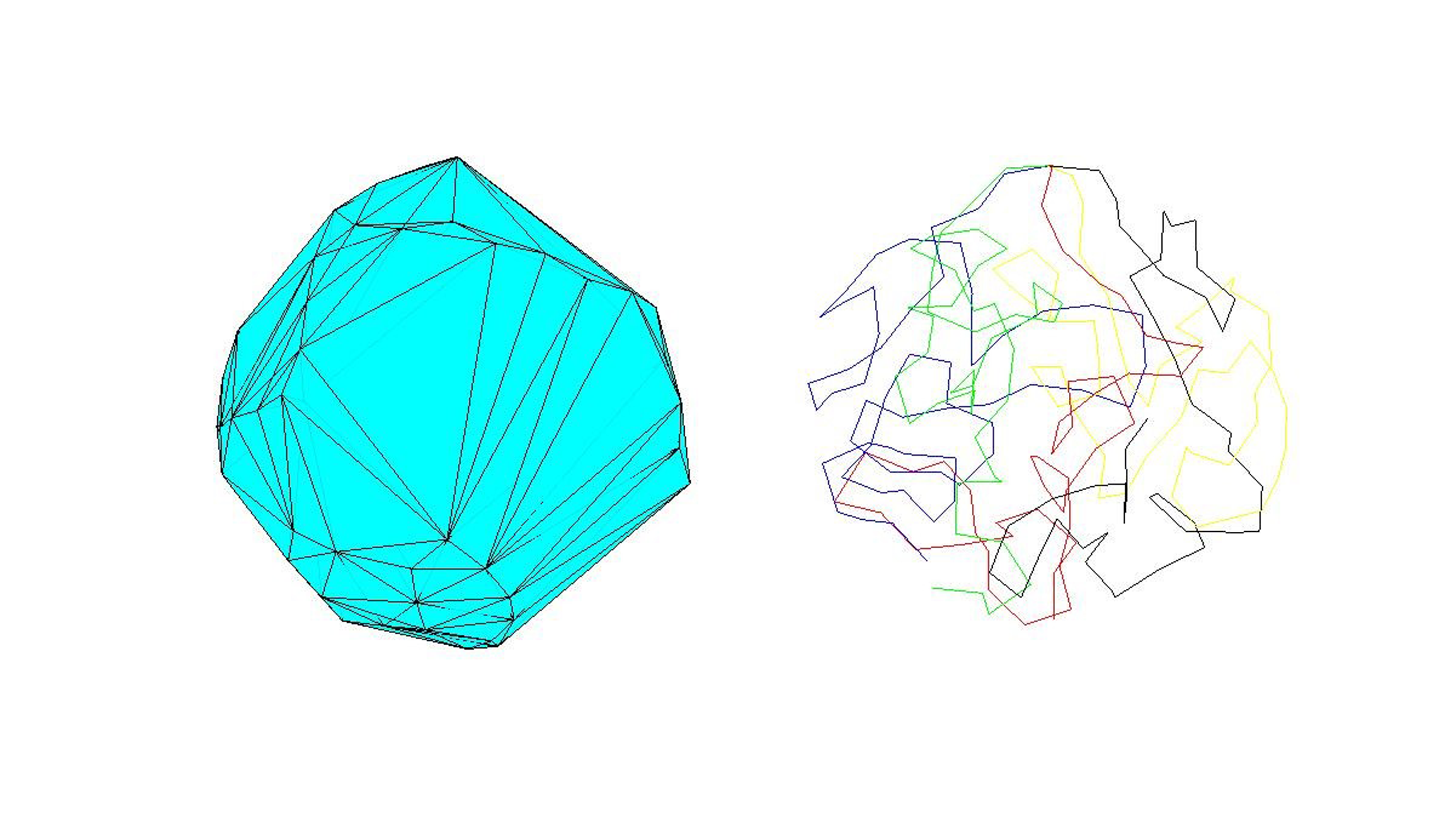

Supplement: Figure S2 — The maximum volume of chromosome convex hull under confinement. The convex hull volume of a chromosome is maximized using a pivot algorithm [2]. Random rotations of chromosome segments are preformed, rejecting those that do not increase the convex hull volume. Iterations are preformed until numerical convergence is achieved. A maximum convex hull for chromosome 3R under confinement (left) is shown next to a model nucleus (right). (TIF) [file pone.0091943.s002.tif]

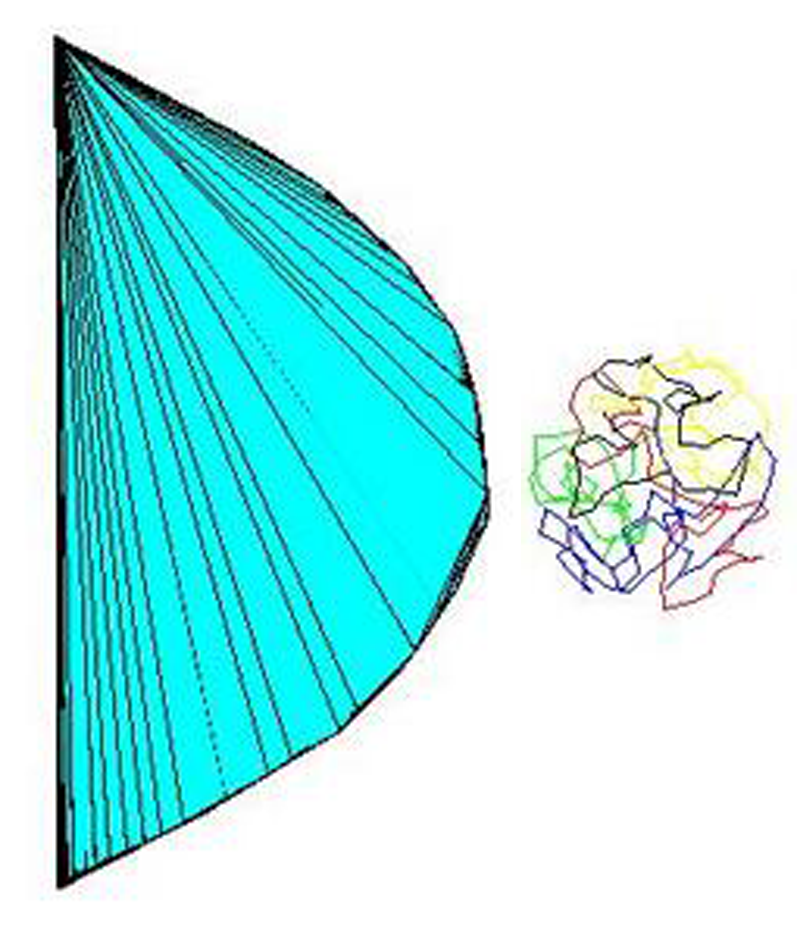

Supplement: Figure S3 — The maximum volume of chromosome convex hull in free space. In free space the maximum convex hull is larger than the entire nucleus. (TIF) [file pone.0091943.s003.tif]

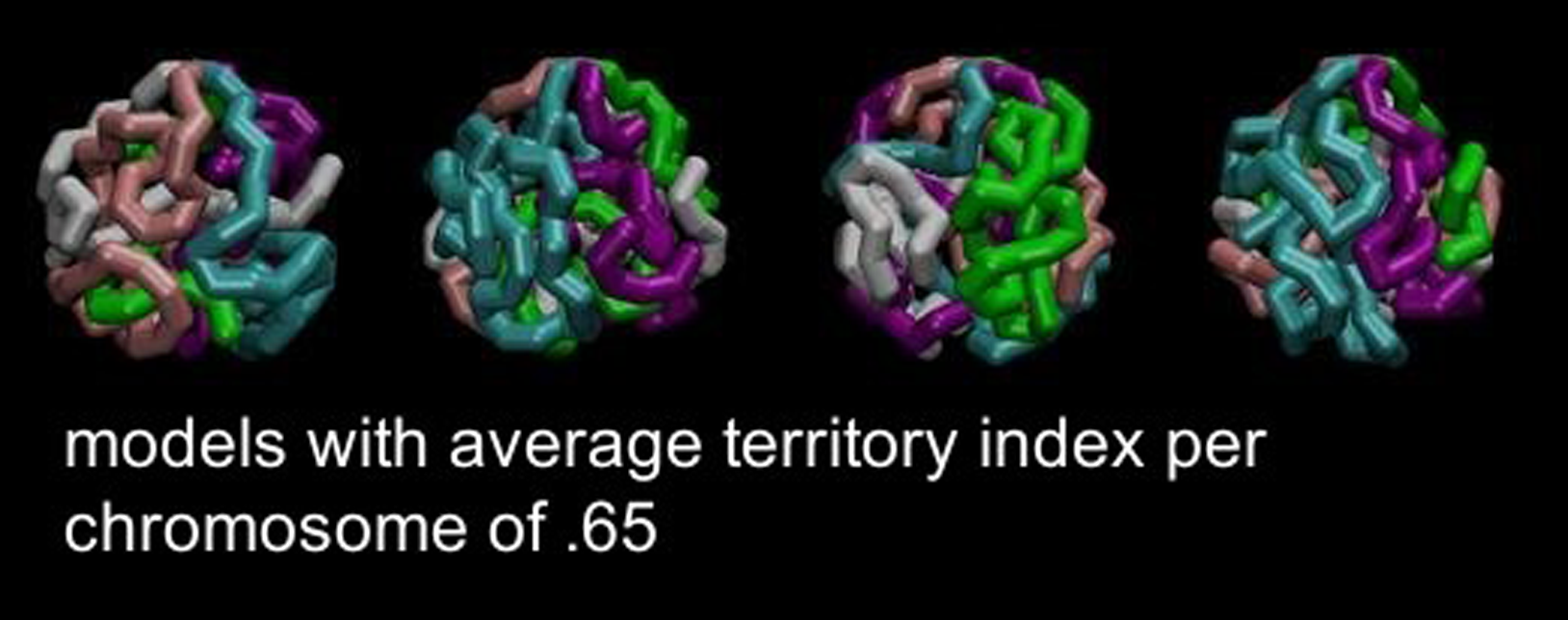

Supplement: Figure S4 — Simulated nuclei with average territory index per chromosome .65. The average territory index per chromosome over all simulated nuclei we generated was .65 (see methods), examples of single model nuclei with this territory index are shown above. The standard deviation of the territory index per chromosome was .04. (TIF) [file pone.0091943.s004.tif]

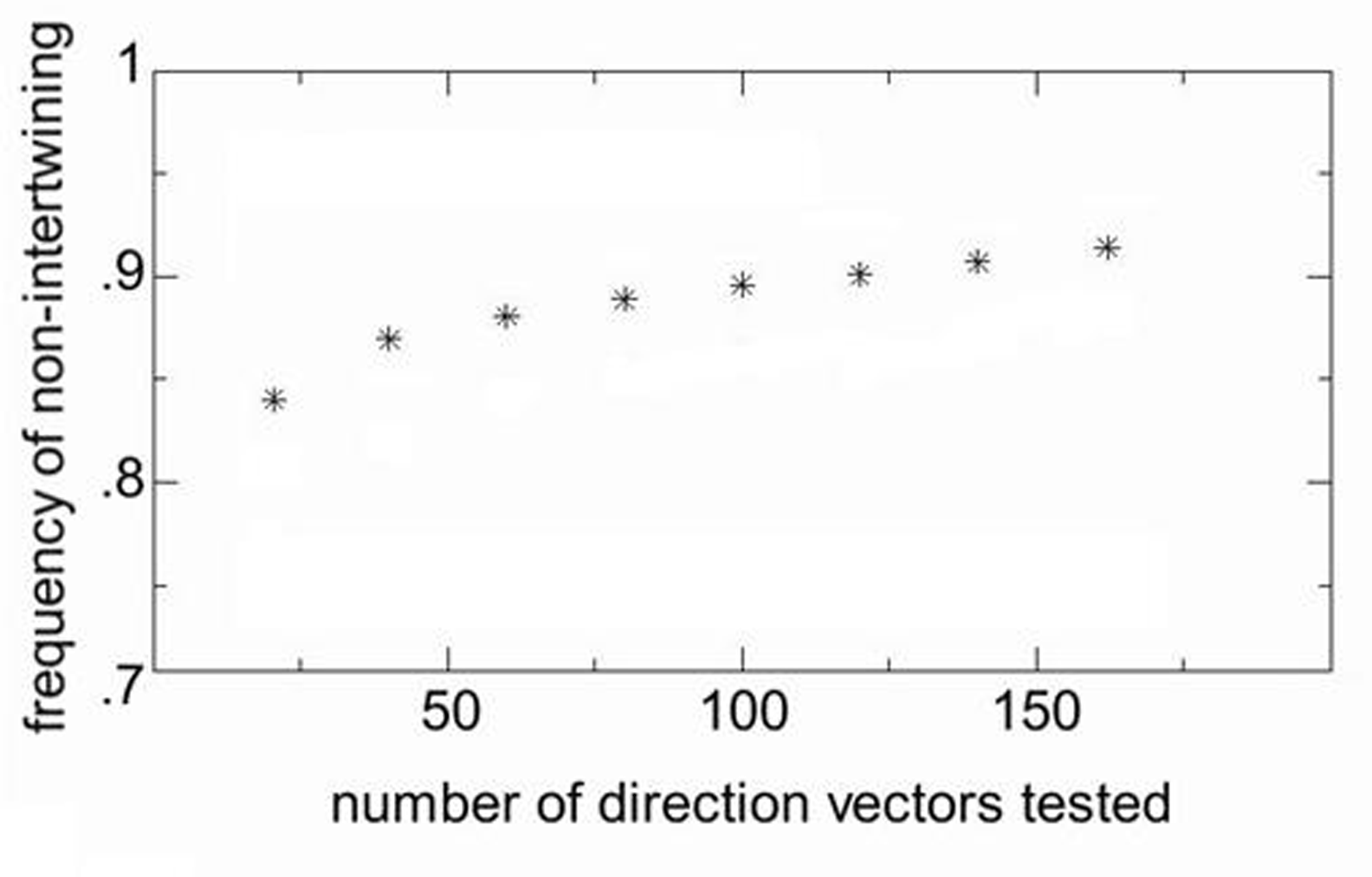

Supplement: Figure S5 — Convergence of the non-intertwining frequency between pairs of chromosomes as the number of test directions for spatial separation is increased. Shown is frequency of non-intertwining depending on the number of direction vectors tested (methods); this suggests that as the number of test directions increases the frequency on non-intertwining chromosomes in our models approaches 95%. (TIF) [file pone.0091943.s005.tif]

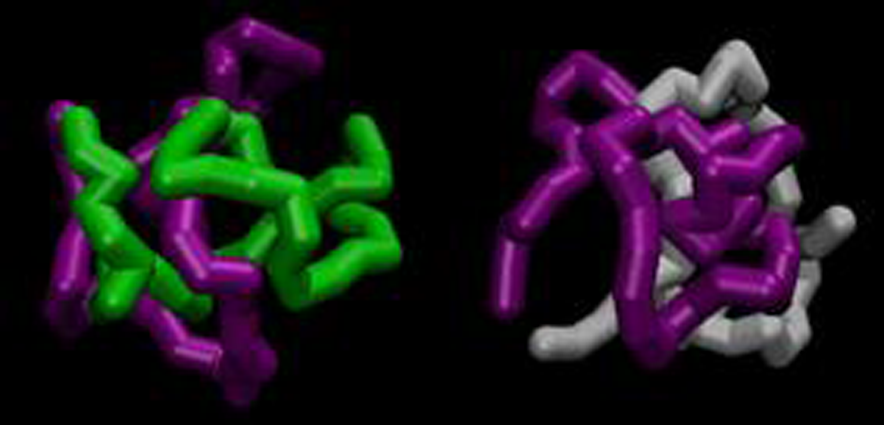

Supplement: Figure S6 — Examples of model chromosomes that intertwine. (TIF) [file pone.0091943.s006.tif]

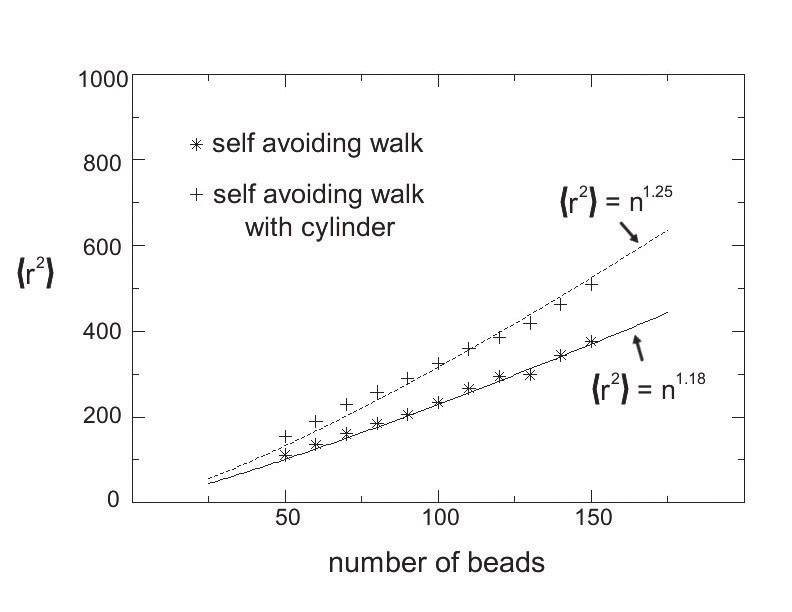

Supplement: Figure S7 — Scaling of self avoiding walks. Each data point represents the square end-end length averaged over 1000 self avoiding walks. This averaging was repeated for self avoiding walks ranging from 50 monomers to 150 monomers. To capture the thickness of the chromosomes a cylinder of excluded volume was placed around the bond between nearest neighbor beads. Scaling with and without this extra excluded volume is shown above. Least square regression lines are shown for each set of points. (TIF) [file pone.0091943.s007.tif]
