# Supplementary material for: Investigation of the Chromosome Regions with Significant Affinity for the Nuclear Envelope in Fruit Fly – A Model Based Approach
Source: PLoS One. 2014 Mar 20;9(3):e91943. doi: 10.1371/journal.pone.0091943 (PMC3961273; doi:10.1371/journal.pone.0091943)
Supplement: Table S1 — Robustness of thresholds to model details. (DOCX) [file pone.0091943.s008.docx]

**Table S1** – **Robustness of thresholds to model details.**

| **Model description** | **$\left\langle\chi+2\sigma\right\rangle$** | **$\left\langle\chi-2\sigma\right\rangle$** |
| --- | --- | --- |
| **96 sets of 24 model nuclei; fully modified SAW (focus of paper)** | **.505** | **.143** |
| **48 sets of 24 model nuclei; fully modified SAW** | **.506** | **.151** |
| **24 sets of 24 model nuclei; fully modified SAW** | **.505** | **.154** |
| **96 sets of 24 model nuclei; unmodified SAW** | **.502** | **.148** |
| **fully modified SAW with BT1 = 1000 and BT2 = 3000** | **.503** | **.143** |
