# Supplementary material for: Investigation of the Chromosome Regions with Significant Affinity for the Nuclear Envelope in Fruit Fly – A Model Based Approach
Source: PLoS One. 2014 Mar 20;9(3):e91943. doi: 10.1371/journal.pone.0091943 (PMC3961273; doi:10.1371/journal.pone.0091943)
Supplement: Table S2 — Robustness of territories and intertwining to model details. (DOCX) [file pone.0091943.s009.docx]

**Table S2** – **Robustness of territories and intertwining to model details.**

| **Model description** | **average territory index** | **Percent non-intertwining** |
| --- | --- | --- |
| **fully modified SAW (focus of paper)** | **.650** | **95%** |
| **unmodified SAW** | **.651** | **95%** |
