# Supplementary material for: Investigation of the Chromosome Regions with Significant Affinity for the Nuclear Envelope in Fruit Fly – A Model Based Approach
Source: PLoS One. 2014 Mar 20;9(3):e91943. doi: 10.1371/journal.pone.0091943 (PMC3961273; doi:10.1371/journal.pone.0091943)
Supplement: Table S3 — Classification of chromosome-nuclear envelope contacts by chromatin type. (DOCX) [file pone.0091943.s010.docx]

**Table S3** - **Classification of chromosome-nuclear envelope contacts by chromatin type.**

| **Chromosome arm** | **NE contact regions** | **LR - Sites of late replication (239 regions) [13]** | **SUUR antibody binding sites in SuUR 4x (280 regions) [13]** | **SUUR antibody binding sites in SuUR 2x (113 regions) [13]** | **Region classification** |
| --- | --- | --- | --- | --- | --- |
| X | 1AB* | 1AB | 1AB | 1AB | IH |
| X | 1EF | 1EF | 1E |  | LR |
| X | 5C | 5C | 5C |  | LR |
| X | 6AB | 6A | 6A | 6A | IH |
| X | 8AB | 8AB | 8B | 8B | IH |
| X | 9A* | 9A | 9A | 9A | IH |
| X | 11C | 11C | 11C |  | LR |
| X | 12E* | 12E | 12E | 12E | IH |
| X | 16D |  |  |  | E |
| X | 17B | 17B |  | 17B | IH |
| X | 18A | 18A | 18A | 18A | IH |
| X | 19DE^ | 19E | 19E | 19E | IH |
| 2L | 21A | 21A | 21A | 21A | IH |
| 2L | 22AB* | 22AB | 22A | 22A | IH |
| 2L | 22D |  |  |  | E |
| 2L | 23C |  |  |  | E |
| 2L | 25EF | 25EF | 25E | 25E | IH |
| 2L | 32A | 32A | 32A | 32A | IH |
| 2L | 32F-33A* | 32F-33A | 32F-33A | 32F-33A | IH |
| 2L | 35AC* | 35BC | 35C | 35C | IH |
| 2L | 36D* | 36D |  | 36D | IH |
| 2L | 37D | 37D | 37D | 37D | IH |
| 2R | 42BC | 42B | 42B | 42B | IH |
| 2R | 45F-46A | 46A | 46A |  | LR |
| 2R | 48EF | 48E | 48EF | 48E | IH |
| 2R | 49CD | 49C | 49D |  | LR |
| 2R | 56F-57A* | 56F-57A | 56F-57A | 56F-57A | IH |
| 2R | 60EF^ | 60F | 60F | 60F | IH |
| 3L | 61AB^ | 61A | 61A | 61A | IH |
| 3L | 62AB |  | 62AB |  | LR |
| 3L | 62D | 62D | 62D |  | LR |
| 3L | 64C* | 64C | 64C | 64C | IH |
| 3L | 67D* | 67D | 67D | 67D | IH |
| 3L | 69BC | 69B |  |  | LR |
| 3L | 70CD*x | 70CD | 70C | 70C | IH |
| 3L | 72AB*x | 72A | 72A |  | LR |
| 3L | 73F-74A | 74A | 74A | 74A | IH |
| 3L | 74E-75A | 75A | 75A | 75A | IH |
| 3L | 79DE | 79DE | 79E | 79E | IH |
| 3R | 83DE* | 83DE | 83DE | 83D | IH |
| 3R | 84A | 84A | 84A | 84A | IH |
| 3R | 86D | 86D | 86D | 86D | IH |
| 3R | 90B |  | 90B |  | LR |
| 3R | 92A | 92A | 92A |  | LR |
| 3R | 97AB^ | 97AB | 97A | 97AB | IH |
| 3R | 98C* | 98C | 98C | 98C | IH |
| 3R | 100BC | 100BC | 100BC | 100BC | IH |
| 3R | 100F* | 100F | 100F | 100F | IH |

* - high frequency NE-associated regions identified at thresholds 0.66-0.63 in Hochstrasser et al 1986 [1] (15 loci)

Unmarked - sub-high frequency NE-contacts at threshold 0.5 (33 loci)

^ - high frequency NE-associated regions identified ALSO in Mathog and Sedat 1989 [9] (4 loci)

x - high frequency NE-associated regions found in Hochstrasser et al 1986 [1] but not in Mathog and Sedat 1989 [9] (2 loci)

E – Euchromatin

LR - Late replicated region (transition stage b/w E and IH)

IH - Intercalary heterochromatin (small-medium size IH bands)

UR IH - Underreplicated intercalary heterochromatin (the largest IH bands)
