# Supplementary material for: Investigation of the Chromosome Regions with Significant Affinity for the Nuclear Envelope in Fruit Fly – A Model Based Approach
Source: PLoS One. 2014 Mar 20;9(3):e91943. doi: 10.1371/journal.pone.0091943 (PMC3961273; doi:10.1371/journal.pone.0091943)
Supplement: Text S1 — Derivation of model parameters and constraints from biological data. (DOC) [file pone.0091943.s011.doc]

**Text S1 - Derivation of model parameters and constraints from biological data**

*Number of chromocenters.* It has been established experimentally that the five chromosome arms of *D. melanogaster* salivary gland share a single common chromocenter in most nuclei ; our models are constructed with a single chromocenter (Figures 1 and 2 main text).

*Chromocenter position.* The chromocenter in *D. melanogaster* salivary gland is always positioned at the nuclear periphery . One bead representing the chromocenter touches the NE in all models (Figure 1 and 2 main text).

*Chromocenter arrangement.* The configuration of the chromocenter, Figure 3, has been described experimentally by recording the order of chromosome arms around the chromocenter. Six different chromocenter arrangements were observed in an experimental set of 22 nuclei ; the number of nuclei satisfying the given arrangement (out of 22) was recorded for each type. In our models the experimental numbers from Figure 3 become frequencies for assigning the corresponding chromocenter arrangement.

*Bead size and chromosome thickness.* The diameter of *D. melanogaster* polytene chromosome can range from 3.1-3.2 microns . Our model uses beads with a diameter of 3.1 microns. To fully capture the thickness of the chromosome fiber we place a cylinder of excluded volume around the bond between nearest neighbor beads (Figure 1 main text). This detail was important for achieving the right nucleus volume to chromosome volume ratio, but was found to have little effect on the scaling of our self-avoiding walks in free space, see below.

*Chromosome length.* The length of the five major chromosome arms have been measured experimentally for *D. melanogaster* salivary gland . Our model incorporates the measured arm lengths by assigning the nearest whole number of beads to each model chromosome arm; X - 45 beads, 2R - 47 beads, 3R – 60 beads, 2L – 46 beads, 3L – 50 beads.

*Chromosome right handedness.* Studies that trace the path of each chromosome arm in *D. melanogaster* salivary gland nuclei have observed a disproportionate amount of right handed twist . This preferred right handedness has been quantified by measuring the triple products of 3 unit vectors (*a, b,* and *c*), tangent to the chromosome path, each spaced 7 microns apart; the triple product, defined as *a·(bxc)*, produces a positive scalar for right handed segments of chromosome and a negative scalar for left-handed segments of chromosome. The distribution of chromosome twist has been measured by calculating the triple product for every set of three vectors formed within a 14 micron window that slides along the path of the chromosome arm. We perform the same analysis during the construction of our SAWs; however, unit vectors that point from the center of one bead to the next nearest neighbor are used in place of unit vectors tangent to the chromosome path. 3.1 microns separate neighboring bead centers; consequently we calculate the triple product of three vectors formed within a 12.4 micron window, rather than a 14 micron window. We enforce right handedness in our simulated chromosomes: during construction of the SAWs, it is twice as likely for a new bead to be accepted if the new triple product formed with this bead is right handed rather than left handed.

*Polytene chromosome persistence length.* Polymer models have estimated a 1.5 micron persistence length for *D. melanogaster* salivary gland polytene chromosomes . A 1.5 micron persistence length means the effective Kuhn length of our model is about twice the persistence length, meeting the condition necessary to build our models as a SAW .

*Nucleus size.* The diameter of *D. melanogaster* salivary gland nuclei has a range of 30–35 microns with an average volume around 19,000 cubic microns. All of our model nuclei have a diameter of 33.5 microns.

*Nucleolus size.* The nucleolus size in our model is based on the measured volume of the nucleolus in *D. melanogaster* salivary glands . Our models use a nucleolus diameter of 7.26 microns (Figure 1 and 4 main text).

*Nucleolus number*. Although some nuclei have more than one nucleolus , we only include one in our models.

*Nucleolus position*. The nucleolus is known to (permanently) associate with the nucleolar organizing region at the base of the X chromosome . In our model the nucleolus position is fixed in space, it touches the base of the single shared chromocenter (Figure 1 and 4 main text).

*Rabl configuration*. It is known that 80% of *D. melanogaster*  polytene chromosomes conform to the Rabl type configuration . This configuration is characterized by the predominant presence of the chromosome telomeres in the nuclear hemisphere opposite the chromocenter. Rabl configuration was enforced in our models by filtering the generated ensembles of nuclei to achieve, in the final ensemble, 80% of telomeres per nucleus in the hemisphere opposite the chromocenter. Specifically, each model nucleus from our ensemble contains 5 chromosome arms. A score (0-5) was assigned to each (unfiltered) model nucleus corresponding to its number of chromsomes in Rabl configuration. All models with a score of 4 or 5 were included in our final (filtered) ensemble. In addition, we have also included a fraction of models with a score of 3, such that the model nuclei in the final ensemble have 80% of telomeres in the hemisphere opposite the chromocenter just like experiment (see figure). The final ensemble contained 96 sets of 24 (2304 total) model nuclei.
