# Supplementary material for: Investigation of the Chromosome Regions with Significant Affinity for the Nuclear Envelope in Fruit Fly – A Model Based Approach
Source: PLoS One. 2014 Mar 20;9(3):e91943. doi: 10.1371/journal.pone.0091943 (PMC3961273; doi:10.1371/journal.pone.0091943)
Supplement: Text S2 — Robustness of threshold used to identify Chr-NE attachments. (DOC) [file pone.0091943.s012.doc]

**Text S2 - Robustness of threshold used to identify Chr-NE attachments**

Our model incorporates *all* experimentally known parameters from *D. melanogaster* polytene chromosomes *with the exception* of introducing specific Chr-NE attachments; in other words, the model is a Null model with respect to chromosome-NE attachment. A threshold, , is then used to identify 48 statistically significant deviations between the null model and experiment which correspond to the regions of Chr-NE attachment (this is described extensively in the main text). We construct the Null model using an equilibrium based self-avoiding walk approach and introduce several modifications in order to recapitulate experiment. Some of these modifications likely introduce non-equilibrium features into our model; however, we stress that the fully modified model contains all the known features of the polytene nucleus from experiment except for specific Chr-NE attachments. For any other model the deviations from experiment would arise from multiple factors, not just the Chr-NE attachments. Regardless, we check that the crucial model conclusion, the statistical thresholds , are robust to the non-equilibrium features that our model contains. Three variations of our SAW approach (also described in the main text) were used for robustness checking of the statistical thresholds, chromosome territories, and chromosome intertwining: *fully modified SAW* – with Rabl configuration, right-handed chirality, and chromocenter arrangement designed to recapitulate all features of experimental nuclei with the exception of Chr-NE attachments; *unmodified SAW* – does *not* introduce Rabl configuration, right-handed chirality, or chromocenter arrangement and is equilibrium to the extent that our chain growing algorithm approximates self-repelling chains (see main text); *fully modified SAW with BT1 = 1000 and BT2 = 3000* – designed to check robustness of model conclusions to the backtracking parameters used in our chain growing algorithm (also see main text). We also checked robustness of the statistical thresholds to the number of models used in the calculation. Results are summarized in table S1 and S2.
