# Supplementary material for: Investigation of the Chromosome Regions with Significant Affinity for the Nuclear Envelope in Fruit Fly – A Model Based Approach
Source: PLoS One. 2014 Mar 20;9(3):e91943. doi: 10.1371/journal.pone.0091943 (PMC3961273; doi:10.1371/journal.pone.0091943)
Supplement: Text S3 — References. (DOC) [file pone.0091943.s013.doc]

**Text S3 – References**

1. Hochstrasser M, Mathog D, Gruenbaum Y, Saumweber H, Sedat JW (1986) Spatial organization of chromosomes in the salivary gland nuclei of Drosophila melanogaster. The Journal of Cell Biology 102: 112-123.

2. Madras N, Sokal AD (1988) The Pivot Algorithm - a Highly Efficient Monte-Carlo Method for the Self-Avoiding Walk. Journal of Statistical Physics 50: 109-186.

3. Hochstrasser M, Sedat JW (1987) Three-dimensional organization of Drosophila melanogaster interphase nuclei. I. Tissue-specific aspects of polytene nuclear architecture. J Cell Biol 104: 1455-1470.

4. E. P. Semionov NKK (1986) Increased number of nucleoli in the salivary gland cells of Drosophila melanogaster under conditions of rDNA dose compensation. Chromosoma 93: 477-482.

5. Ostashevsky J (2002) A polymer model for large-scale chromatin organization in lower eukaryotes. Mol Biol Cell 13: 2157-2169.

6. Grosberg AY KA (1994) Statistical physics of macromolecules. New York: AIP.

7. Tark-Dame M, van Driel R, Heermann D (2011) Chromatin folding--from biology to polymer models and back. Journal of cell science 124: 839-845.

8. Zhimulev IF (1996) Morphology and structure of polytene chromosomes. Advances in Genetics, Vol 34 34: 1-490.

9. Mathog D, Sedat JW (1989) The Three-Dimensional Organization of Polytene Nuclei in Male Drosophila melanogaster With Compound XY or Ring X Chromosomes. Genetics 121: 293-311.

10. Hochstrasser M (1987) Chromosome structure in four wild-type polytene tissues of Drosophila melanogaster. Chromosoma 95: 197-208.

11. Kaufmann BP (1937) Nucleolus-Organizing Regions in Salivary Gland Chromosomes of Drosophila Melanogaster. Cell and Tissue Research 28: 1-11.

12. Hochstrasser M, Sedat JW (1987) 3-Dimensional Organization of Drosophila-Melanogaster Interphase Nuclei. 1. Tissue-Specific Aspects of Polytene Nuclear Architecture. Journal of Cell Biology 104: 1455-1470.

13. Zhimulev IF, Belyaeva ES, Makunin IV, Pirrotta V, Volkova EI, et al. (2003) Influence of the SuUR gene on intercalary heterochromatin in Drosophila melanogaster polytene chromosomes. Chromosoma 111: 377-398.
